# Toward self-regulated learning: effects of different types of data-driven feedback on pupils’ mathematics word problem-solving performance

**DOI:** 10.3389/fpsyg.2024.1356852

**Published:** 2024-10-01

**Authors:** Jun Huang, Yining Cai, Ziying Lv, Yuanbo Huang, Xiao-Li Zheng

**Affiliations:** ^1^School of Teacher Education, Central China Normal University, Wuhan, China; ^2^Department of Educational Technology, Wenzhou University, Wenzhou, China; ^3^Office Of the Registrar of Wenzhou-Kean University, Wenzhou, China

**Keywords:** interactive e-book, self-regulated learning, data-driven feedback, mathematics, word problem solving

## Abstract

**Introduction:**

Mathematical word problems refer to word problems where the information that is presented needs to be integrated, typically into a mathematical formula, to arrive at a solution to the problem. When solving mathematics word problems, elementary school students often have difficulties improving their performance due to a lack of self-regulated learning (SRL). However, SRL can be developed by adopting an appropriate teaching approach which offers quantitative feedback or learning prompts. With the sophistication of interactive and data-driven feedback technology, it is possible to provide timely and personalized strategies for promoting students’ SRL.

**Methods:**

In this study, an interactive e-book editing platform was used to design self-regulation-level-based feedback(SRLF) and task-level-based feedback(TLF) teaching models, which were respectively conducted in two similar fifth-grade classes for the mathematics word problem solving lessons.

**Results:**

Using ANCOVA and repeated ANOVA, this study found that (1) the SRLF had a remarkably greater impact on elementary school students’ mathematics word problem-solving performance than the TLF, with a partial *η*^2^-value of .107; (2) In the short period of time, there was no significant difference between the two kinds of feedback on the learners’ SRL. The TLF was slightly superior to the SRLF, especially in terms of total self-regulated learning scores and cognitive strategies; (3) The TLF had a significant interaction effect on self-regulated learning and cognitive strategies, respectively with a partial *η*^2^-value of .059 and .056.

## 1 Introduction

In recent decades, the development of mathematical problem-solving skills has become an essential teaching goal in mathematics education (Panaoura, 2012). However, according to Harskamp and Suhre (2006), teachers still have trouble teaching pupils how to solve math problems using the proper methods. The reason for students’ failure to solve mathematical word problems is not a lack of mathematical knowledge but a lack of self-regulated learning (SRL) in the process of solving the problems (Kizilcec et al., 2017). Therefore, it is important to provide appropriate approaches to facilitate the development of SRL in the learning process in order to improve students’ mathematical problem-solving ability. SRL is a crucial component of achieving academic success (Schunk and Zimmerman, 2007; Daniela, 2015; Demssie et al., 2023) as it engages students in modifying their learning behavior during curriculum learning in order to improve their learning outcomes and performance (Vilkova, 2022). In addition, previous studies demonstrated that efficient feedback and suggestions on behavior learning such as SRL methods are crucial for SRL, and significantly correlate with student achievement (Algayres and Triantafyllou, 2020; Li and Shein, 2022). However, it is difficult for teachers to understand how feedback and behavioral suggestions should be provided during teaching activities to help students improve their learning performance (Nicol, 2010).

To address this issue, researchers have carried out several studies that have supported students’ SRL and enhanced their learning outcomes. Based on learning analytics, Garcia et al. (2018) developed websites for feedback and reflection to assist students in SRL phases such as goal-setting, monitoring and reflection. In addition, some studies have collected students’ answers through questionnaires, and given corresponding feedback to verify the impact of goal setting, task planning and learning motivation on students’ SRL (Kizilcec et al., 2017; Manzanares et al., 2017). However, the above methods only measured SRL using self-reported questionnaires (Alonso-Mencía et al., 2021), and did not use personalized feedback methods based on students’ specific learning performance. Moreover, in some studies, the performance prediction output was used as feedback, but it did not provide evidence for prediction or any meaningful insights or practicable information, which means that students did not receive any feedback on their learning performance (Baneres et al., 2019; Cano and Leonard, 2019; Mubarak et al., 2022; Yang and Ogata, 2023).

In order to further differentiate personalized feedback, based on the feedback content, Hattie and Timperley (2007) noted that there are four categories of feedback: task, task processing, self-regulation and self-level. TLF usually gives information about a specific task, mainly referring to the learning task (Kluger and DeNisi, 1996; Petrovic et al., 2017) and aims to provide simple hints or detailed explanations according to the level of difficulty (Timmers et al., 2015). Task-processing-based feedback is more specific to the processes underlying tasks or to related and extended tasks. Such feedback concerns information about relations in the environment, relations perceived by a person, and relations between the environment and the person’s perceptions, providing more specific cues and scaffolding for the task processing (Balzer et al., 1989). SRLF can offer personalized scaffolds (e.g., feedback provided by artificial intelligence) to catalyze self-regulation, guide student learning, enhance self-efficacy, and contribute to higher student achievement to support students in successfully completing learning tasks (Butler and Winne, 1995; Afzaal et al., 2023). Self-level-based feedback is usually unrelated to the learning task (Faber et al., 2017). Examples of self-level feedback in teaching often include “You are a good student.” Table 1 compares the application cases and effects of the four types of feedback.

**Table 1:** Comparison of the application cases and effects of the four types of feedback.

Feedback type	Application cases	Learning achievement	Self-regulated learning
Task-level-based feedback	Educational programming game-based learning (Mao et al., 2024)	Overall (+) Learning achievement (+) Learning engagement (−)	NA
	English writing (Teng, 2020)	Overall (+)	NA
	Literacy game-based learning (Vasalou et al., 2021)	Overall (+)	NA
	Mathematic (Guo et al., 2019)	Overall (+)	Overall (+)
Task-processing-based feedback	Higher education (Zepeda et al., 2023)	NA	NA
Self-regulation-level-based feedback	Asynchronous community college course (Gaul and Kim, 2020; Shams, 2023)	Overall (+)	NA
	Second language (L2) writing (Sherafati and Mahmoudi Largani, 2023; Yang and Zhang, 2023)	Overall (+)	Overall (+)
	Mathematics Learning (Labuhn et al., 2010)	Overall (+)	Overall (+) Self-evaluation (+)
Self-level-based feedback	High school courses (Guo, 2020)	NA	Overall (+)

The sign “+” means positive group difference; “–” means negative group difference; “NA” means no available results.

Hattie and Timperley (2007) stated that self-level is the least effective form of feedback. In comparison, self-regulation-level and task-level feedback are powerful in terms of deep processing and mastery of tasks. In other words, both types of feedback are able to contribute to improving students’ learning performance. At the same time, TLF is powerful when the task information is subsequently useful for improving strategy processing or enhancing self-regulation (which it too rarely does). In summary, the two most commonly applied forms of feedback on student learning outcomes are TLF and SRLF. Hence, this study focused on these two types of feedback. Although some researchers have recognized the importance of feedback in SRL (Cavanagh et al., 2020; Afzaal et al., 2021), there are still many questions about how to design a data-driven feedback approach, and differences among their effects on SRL and mathematical problem solving have not been explored in depth.

With the sophistication of interactive and data analytic technologies, data-driven feedback teaching has gained widespread attention because it is possible to give specific feedback and guidance according to students’ actual learning status. Therefore, to enhance the effectiveness of data-driven personalized feedback teaching, it is important to provide students with a SRL approach. In this study, TLF and SRLF approaches were proposed to develop students’ SRL. A data-driven feedback-based teaching system (DDFTS) was developed according to the proposed approach to enable students to determine the learning goals, engage in learning along with planning, monitoring and evaluating their own learning performance, and make reflections accordingly. Data-driven feedback is the prediction of a student’s academic performance with an explanation of the underlying reasons for the prediction, and the automatic provisions of data-driven, intelligent suggestions. Moreover, an empirical study was administered to evaluate the effectiveness of different types of data-driven feedback on students’ learning achievement and SRL. In this study, the main research questions were addressed as follows:

*RQ1*: How does the impact of TLF compare to SRLF in promoting self-regulated learning?

*RQ2*: How does the impact of TLF compare to SRLF in promoting mathematics word problem-solving performance?

## 2 Literature review

This section comprises three parts. The first part outlines the definition and framework of SRL, the second concentrates on introducing the personalized feedback method for SRL and its interconnection with mathematics teaching, and the last part provides an overview of the development of mathematics problem solving.

### 2.1 Self-regulated learning

SRL is considered as an underlying learning process that enhances students’ learning motivation and reflects on their learning process, thereby contributing to their learning (Michalsky and Schechter, 2013). Through SRL, students can develop a deep understanding of complex issues during the learning process (Labuhn et al., 2008; Panadero and Järvelä, 2015; Tian et al., 2018). Meanwhile, their behaviors and attitudes consistent with SRL also contribute to their self-confidence (Artino and Jones, 2012).

**Figure 1:**
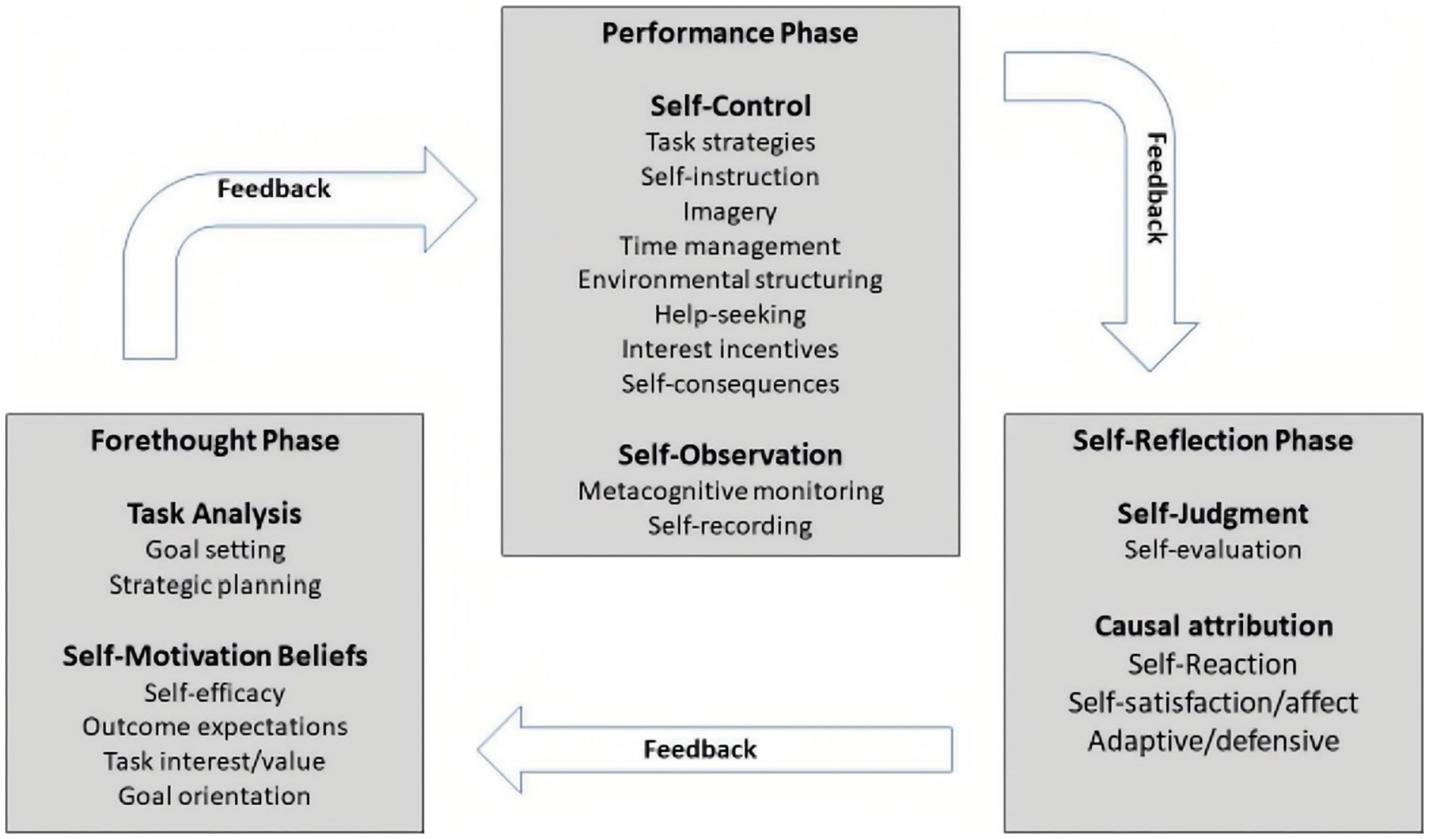
Zimmerman’s SRL model.

SRL specifically refers to the process whereby learners spontaneously and proactively set goals, employ various strategies, and monitor and evaluate their own behavior and learning outcomes to achieve their learning goals (Zimmerman, 2008). Based on empirical research and a social-cognitive framework, Zimmerman (2002) developed a cyclical framework of academic SRL that consists of various processes that learners purposely use to manage their behaviors, cognition, emotions, and environment to attain their personal goals (Figure 1). This framework revealed that SRL includes three stages: the forethought phase, the performance phase, and the self-reflection phase. In the forethought phase, students should analyze the learning tasks and set specific learning goals and strategies to achieve these goals. The performance phase refers to students learning based on learning strategies, and trying their best to achieve their learning goals (Zimmerman, 2002). During these processes, students could be aware of their performance with regard to certain learning goals, and need to monitor the appropriate learning strategies in order to achieve their goals. The self-reflection phase indicates how students evaluate the correlations between their learning results and learning strategies in order to determine the effectiveness of the learning strategies. This SRL model has been empirically researched and applied to the learning of various disciplines, such as mathematics (Chen and Zimmerman, 2007).

SRL has been a hot topic of research in the field of learning theory, and researchers have conducted a great deal of research on how to promote it. Regarding strategies to develop SRL competence, most of the existing research used motivational strategies (such as attribution training, behavioral control, etc.), metacognitive strategies (such as planning, monitoring, evaluation, etc.), and cognitive strategies (such as organizational information strategies), detailed strategies, and various problem-solving strategies to intervene in students’ SRL process. Depending on the respective underlying model of SRL, the interventions emphasize different aspects of the learning process (Dignath and Büttner, 2008). According to Timmons et al. (2016), early interventions to promote students’ SRL are delivered through cognitive-behavioral modification programs or direct instruction strategies. Innovations in later classroom intervention include changes to traditional classroom arrangements to build learners’ responsibility and independence. This is consistent with Bae and Kwon (2021), who found in recent years that metacognitive interventions have shifted from strategic training methods to creating social environments to support metacognition. Sufficient empirical studies have indicated that SRL can be significantly improved after intervention (Narciss et al., 2007; Chen and Chien-Yuan, 2019). Despite these studies, research in this field is still needed to clarify the effectiveness of various interventions (Schunk, 2005).

### 2.2 A personalized feedback approach to self-regulated learning

#### 2.2.1 Feedback

Feedback is information provided by an agent regarding aspects of one’s performance or understanding (Hattie and Timperley, 2007). Researchers have conducted extensive studies on the effectiveness of feedback and have proven that it can effectively promote students’ achievement (Wisniewski et al., 2020) and their SRL (Devolder et al., 2012). Regarding the content of different types of feedback, researchers have compared the effects of praise, punishment, rewards and corrective feedback on learning effects. The results showed that corrective feedback is the most effective. Regarding different feedback channels, researchers have shown that the combined effect of video/audio and computer-assisted feedback is better (Wisniewski et al., 2020). Meanwhile, Hattie and Timperley (2007) investigated the timing of feedback (immediate/delayed) and the valence (positive/negative feedback), reporting inconsistent results. However, few studies have paid attention to the effect size between TLF and SRLF.

#### 2.2.2 Personalization and personalized feedback

Personalized learning is an ambitious promise of computer-assisted education (Kabudi et al., 2021). Through personalized digital learning, instructors are empowered to tailor their teaching methods to address the specific needs and characteristics of individual students (Murphy, 2019; Hwang et al., 2020). Feedback plays a critical role in personalized learning scenarios (Maier and Klotz, 2022). Currently, research focuses on the application of personalized dynamic feedback supported by a computer environment in SRL. Personalized dynamic feedback enables interactive assessment of students’ learning performance and provides feedback as needed (Kim and Hannafin, 2011), which is an important method for developing students’ SRL.

#### 2.2.3 Data-driven personalized feedback

Personalized dynamic feedback is inseparable from the support of data. The data-driven personalized feedback teaching paradigm has emerged and is widely used in schools as a result of the advancement of information technology (Kapoor et al., 2023; Söderström, 2023). The data-driven personalized feedback approach includes two processes: precise analysis of questions from learning process data and precise personalized feedback with the guidance of data analysis. Feedback is an important component of formative assessment, which is defined as “all activities which are performed by teachers and students [that] can provide information as feedback to modify the teaching and learning activities they engage in” (Black and Wiliam, 1998). Therefore, feedback is an important part of SRL. Computer-based feedback can be delivered to students more quickly than teacher-based feedback, and different students can receive feedback at the same time. However, research has shown that the content of feedback has a greater effect on students than the method of feedback (teacher or computer) (Lipnevich and Smith, 2009). In other words, the content of the feedback is the key element that determines the final learning effects for students. Therefore, this study focused only on how to analyze data and design different feedback content rather than feedback methods in a computer-assisted environment to further improve the precision of the teaching design.

#### 2.2.4 Data-driven personalized feedback and mathematics teaching

Previous studies have shown that data-driven personalized feedback teaching has a significantly positive effect on students’ mathematics learning (Ramey et al., 2016) and is an effective way to improve the effectiveness of mathematics teaching. Sleeman and Krawczyk (2021) found that the data-driven personalized feedback approach to SRL can significantly promote elementary school students’ learning of mathematics multiplication, and can effectively improve students’ problem-solving speed through an empirical study. Kapoor et al. (2023) designed a data-driven feedback approach for mathematics. Students completed exercises through the Zoom and WhatsApp platforms and received feedback on their grades. The results showed that this model can significantly improve primary school students’ ability to calculate mixed addition and subtraction. In addition, Söderström (2023) designed metacognitive feedback scaffolding and heuristic feedback scaffolding in a computer-assisted environment to provide students with personalized feedback on the process of solving mathematics word problems, and the results illustrated that these two scaffolds helped students understand the context of mathematics problems and improve their learning performance.

In sum, studies on personalized feedback approaches to SRL in elementary school mathematics have mainly focused on the validation of single or dual feedback models without focusing on the comparative learning effects of different feedback approaches. Moreover, most studies regarded learning performance as the main indicator of the feedback approach, with fewer studies involving other aspects of students’ learning performance (Nicolaou et al., 2009) such as SRL, which plays an important role in academic success. Therefore, it is necessary to explore how the data-driven feedback approach affects SRL.

#### 2.2.5 Personalized feedback approaches to SRL

Personalized dynamic feedback enables interactive assessment of students’ learning performance and provides feedback as needed (Kim and Hannafin, 2011); it is an important method for developing students’ SRL. Schoppek and Tulis (2010) developed an adaptive learning system that provided personalized diagnosis, problem selection, and immediate feedback to support students’ SRL, and showed that a personalized feedback approach embedded in the adaptive learning system made a substantial contribution to the improvement of mathematics word or arithmetic problem-solving. The adaptive feedback learning is mainly adapted to the learner’s knowledge level, and the amount of feedback is the main feature of feedback variation in the adaptation process (Maier and Klotz, 2022). Afzaal et al. (2023) designed an artificial intelligence-supported learning website to provide personalized feedback and suggestions with a data-driven approach, and the results showed that students’ course grades and SRL were significantly improved.

In sum, the personalized feedback approach supports students’ SRL by monitoring their learning process and providing intelligent behavioral prompts and timely dynamic feedback, which is an effective way to improve learning performance and SRL. However, to date, the impact of the personalized feedback-based approach on mathematics word problem-solving performance and SRL has not been deeply explored. Hence, this study designed the TLF and the SRLF approaches to investigate their effects on mathematics word problem-solving performance and SRL with the intent to provide insights for future research and practice.

### 2.3 Mathematics problem solving

Mathematics problem solving refers to the use of mathematics concepts grasped by students to solve problems (Muis et al., 2016). It is considered to be the core of teaching and learning mathematics (Hasibuan et al., 2018) and helps students make associations between the mathematics concepts learned and real-world applications (Verschaffel et al., 2010). Researchers have conducted a number of empirical studies on the classification of math problem-solving difficulties (Mayfield and Chase, 2002), identifying factors that influence the problem-solving processes of elementary school students (Öztürk et al., 2020; Vondrová, 2022; Herbert and Williams, 2023), designing the problem-solving teaching process (Masingila et al., 2018; Copur-Gencturk and Doleck, 2021), and so on. In sum, the ultimate objective of the above studies was to develop mathematics problem-solving skills.

For elementary school students, mathematical problem solving often includes solutions and exercises of mathematical word problems, which require students to extract useful numerical information from descriptive text of the problem context and to perform arithmetic operations on it (Verschaffel et al., 2020). Several previous studies have shown that feedback-based teaching can help students improve their performance in mathematics word problems (Muis et al., 2016; Faber et al., 2017; Söderström, 2023). Researchers have carried out a large number of studies on the effectiveness of the personalized feedback-based approach on mathematics word problem-solving performance for elementary school students.

Orosco et al. (2013) designed a dynamic formative assessment strategy for mathematics in which teachers provided the feedback associated with students’ reading and language comprehension levels, and assessed its effectiveness on word problem solving for Latino English learners. Results showed that this feedback-based approach could significantly improve students’ mathematics word problem-solving performance. With the development of artificial intelligence technology, the subject of implementing feedback teaching has gradually changed from teachers to computers, and digital formative assessment tools have become the main implementation vehicles (Xu et al., 2021). Faber et al. (2017) designed a digital formative assessment tool that provides student feedback, teacher feedback, and personalized homework functions, and verified its effects on elementary school students’ mathematics word problem-solving performance and mathematics learning motivation. The results showed that the tool had a positive impact on students’ mathematics word problem-solving performance and learning motivation. To sum up, the current vehicles for personalized feedback-based approach are driven mainly by digital formative assessment tools, and the effectiveness of formative assessment tools largely depends on their feedback content. Thus, it is very important to design feedback tools to support the formative assessment process.

## 3 The personalized feedback approach for mathematics word problems

This section is divided into four parts; 3.1 and 3.2 present the two levels of feedback approach, where 3.1 describes the TLF approach, and 3.2 describes the SRLF approach. Based on the previous two subsections, in this study we proposed a data-driven feedback-based teaching system, as shown in 3.3. In order to verify the effectiveness of this system, the teaching activities of mathematical application problems based on this system were designed and implemented, as shown in 3.4.

### 3.1 A task-level-based feedback approach

**Figure 2:**
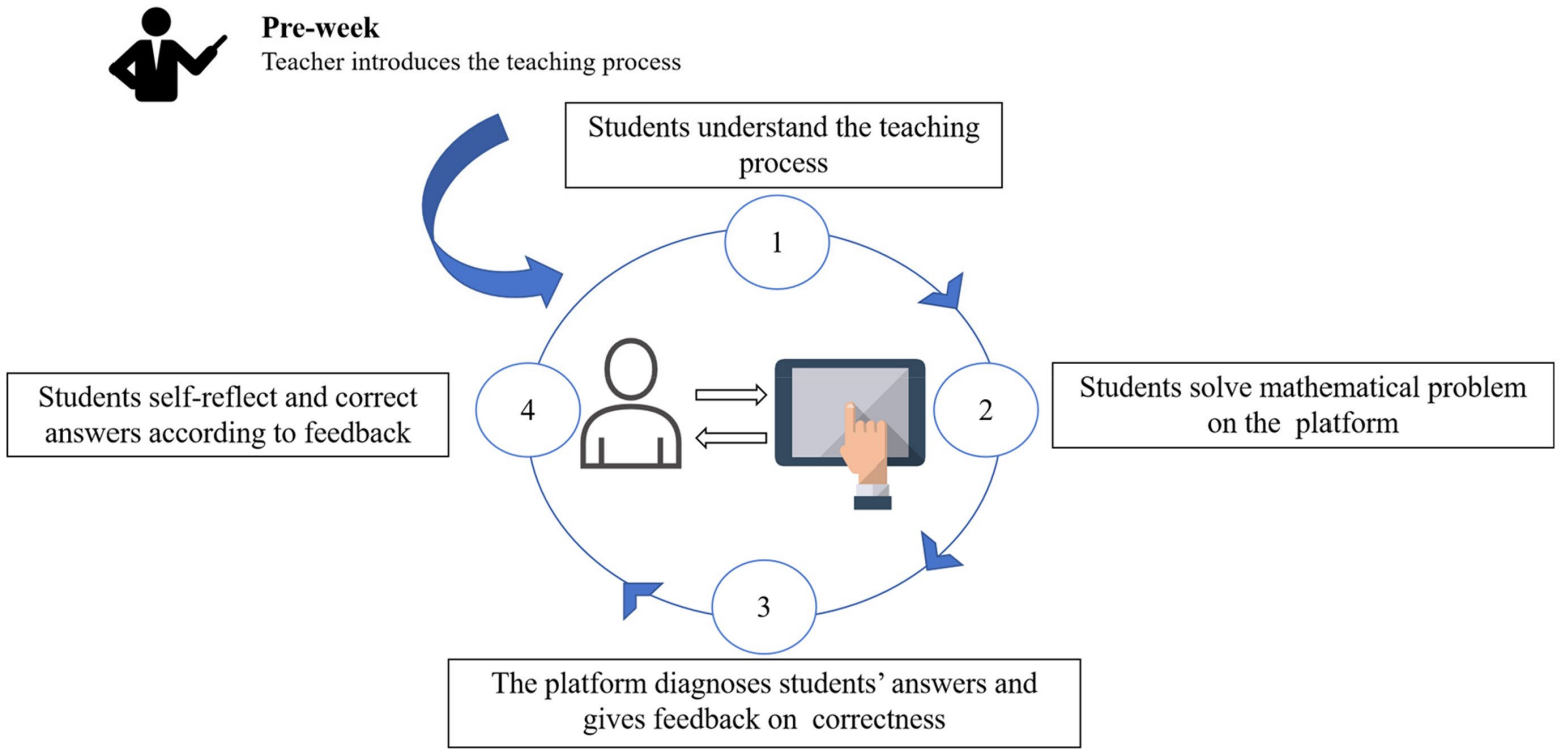
A task-level-based feedback approach.

This study used the interactive e-book editing platform to implement a TLF approach. All teaching activities were implemented on the interactive e-book editing platform. Compared to the SRLF approach, the TLF approach provided feedback about the rate of correctness. The specific approach is shown in Figure 2.

First, the teacher introduced the teaching process before learning so that students could understand it. Second, students solved mathematical problems and submitted their answers via the interactive e-book editing platform. After that, the platform automatically corrected the answers and presented feedback on the correctness of their answers to the students. Finally, students self-reflected and corrected their responses based on the feedback results.

### 3.2 A self-regulation-level-based feedback approach

Similar to the TLF approach, this study also used an interactive e-book platform to implement a SRLF approach which provided specific guidance on the cause of the errors, so that students could reflect and correct the answers according to the feedback.

**Figure 3:**
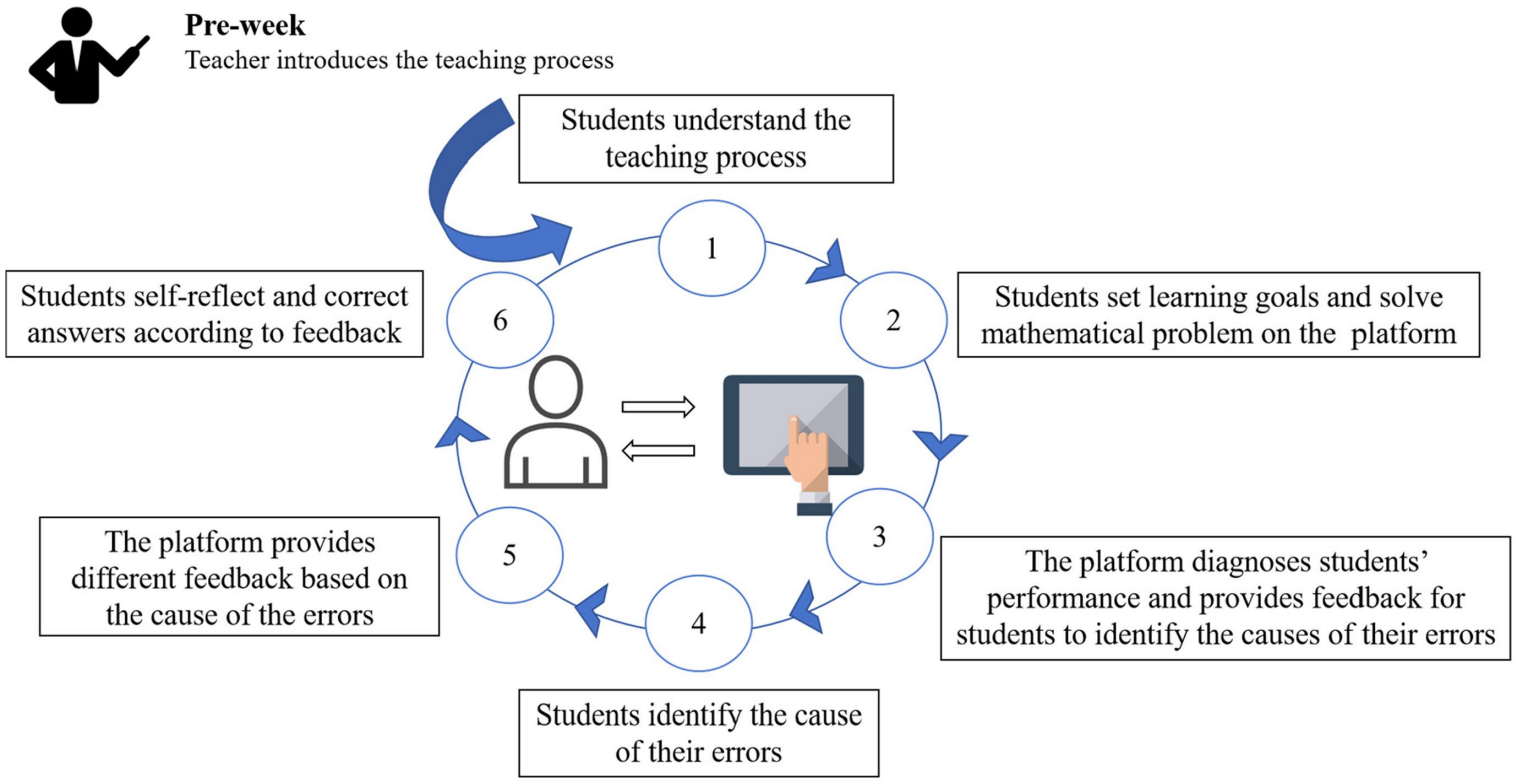
The self-regulation-level-based feedback approach.

The teacher introduced the teaching process before learning so that students could understand the process. After that, students set learning goals and solved mathematical problems on the platform. The platform automatically diagnosed students’ performance and provided feedback for them to identify the causes of their errors. Students identified the causes of their errors based on the feedback from the interactive e-book editing platform. The platform provided different feedback based on the cause of the errors. Students self-reflected and corrected their answers based on the feedback results. The specific approach is shown in Figure 3.

### 3.3 The design of the data-driven feedback-based teaching system

Various feedback teaching tools have been widely used in teaching to support self-regulated learning (Sung et al., 2016). However, most researchers have focused on a single tool (Yang et al., 2018), while there has been a lack of attention to the impact of data-driven feedback-based teaching systems on SRL. Therefore, in order to validate the effectiveness of the personalized feedback-based approach, in this study we constructed a data-driven feedback-based teaching system (DDFTS) to support students’ SRL in the process of mathematics learning.

**Figure 4:**
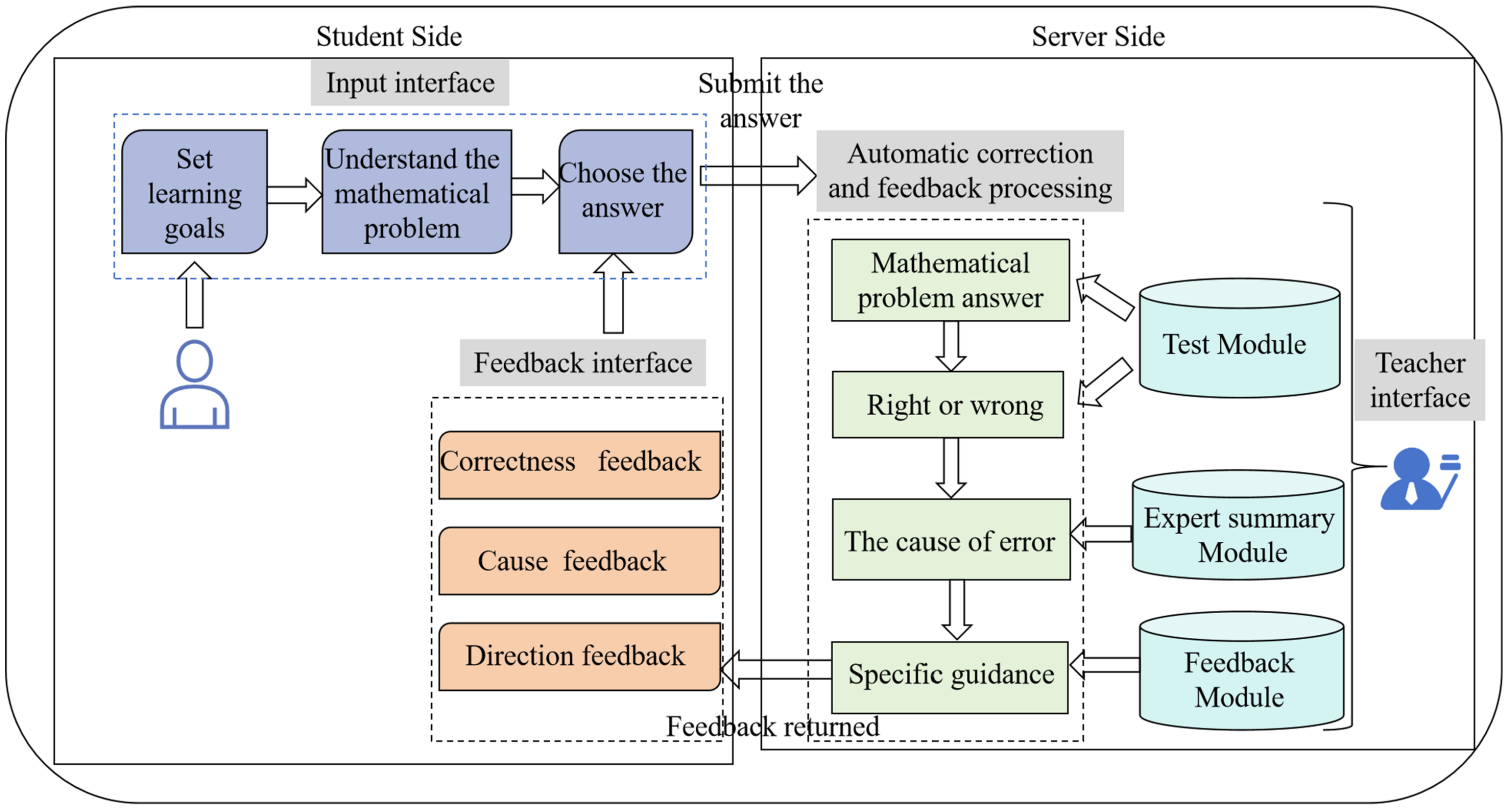
The framework of the DDFTS system.

The design principle of the DDFTS system is based on the three-phase model of SRL (Zimmerman, 2002), in which learning consists of the forethought, performance, and self-reflection phases. The structure of this system is shown in Figure 4.

The student side consists of the input interface and the feedback interface. On the input interface, students can set learning goals, which is the forethought phase. The students’ editing page on the interactive e-book platform is shown in Supplementary Figure 1. The interface for setting learning goals is shown in Supplementary Figure 2. Students used this platform to flip pages, add sticky notes, set learning objectives based on options, and so on. After that, students tried to understand mathematical problems, chose the answer and submitted it, which was the performance phase. On the feedback interface, students could receive three different types of feedback: accuracy, cause and direction feedback. The interface for cause feedback is shown in Supplementary Figure 3. When the feedback was returned, students determined the cause of the error and reflected based on the feedback in order to better understand how to solve the mathematical problems, which was part of the self-reflection phase.

The server side consists of automatic correction and feedback processing. The teacher interface consists of a test module, an expert summary module, and a feedback module. In the test module, teachers can plan online learning activities, including setting learning goals, organizing the mathematical problem test and setting up the mathematical problem’s correct answer. In the expert summary module, teachers can summarize the cause of error for the mathematical problem test. In the feedback module, teachers can give specific guidance based on the cause of error. Then, teachers upload these course materials to the platform. The teacher’s edit interface is shown in Supplementary Figure 4. Teachers can preview the courseware, insert test modules and pictures, and so on.

### 3.4 Mathematics word problem-solving activities based on data-driven personalized feedback

In this study, the teacher introduced the teaching process before teaching, and two groups of students completed a pretest with mathematics word problems, and submitted their answers via the interactive e-book editing platform, which automatically corrected the test questions and gave feedback to the students, including on the accuracy of their responses. The feedback interface of automatic correction on the interactive e-book editing platform is shown in Supplementary Figure 5. This interface shows the automatic correction feedback. Supplementary Figure 6 shows the direction of the feedback interface on the interactive e-book editing platform, giving an example of a mathematics question. Finally, students reflected and corrected their answers according to the feedback.

In the formal teaching stage, the control group continued to use the TLF approach. The students in the experimental group used the SRLF. According to the results of the pretest and the interviews with the students, this study attributed the reasons for students’ errors in solving mathematics word problems to “failure to understand the meaning of the problem,” “unclear known data and problem,” and “wrong calculation.” The above reasons were presented as options on the interactive e-book platform, and students could choose multiple items according to their situation and click on them to jump to the corresponding feedback scaffolding for self-reflection and correction. The self-reflection activities-based feedback on the interactive e-book platform is shown in Supplementary Figure 7. On this interface, students could self-reflect and correct their answers on sticky notes based on the feedback they received.

## 4 Research methodology

This section is divided into three parts. The basic information of the participants in this study is introduced in 4.1, while the experimental procedure and instruments used in this study are described in 4.2 and 4.3.

### 4.1 Participants

The 69 participants (32 female and 37 male students) were from two fifth-grade classes of a public elementary school (the best primary school in W city), with ages ranging from 11 to 13 years old. Their parents’ social status was at an upper-middle level. The average age of the participants was 11.73 years (min: 11, max: 13). They participated in a 4-week mathematics course that focused on mathematical problem solving.

The experimental group, including 35 students, learned using the SRLF approach. On the other hand, the control group with 34 students learned with the TLF approach. Before teaching, they were informed of the learning instructions and test, and signed a consent form to participate in a series of teaching activities along with their parents’ approval. After comparing the means of the two groups, we found that there was no significant difference. Therefore, one of the classes was designated as the experimental group and the other students were assigned to be the control group.

### 4.2 Experimental procedure

This research was reviewed and approved by the Institution Review Board of the affiliated institution (Code number: WZU-2023-099). The experiment was conducted in this study to investigate the effects of the personalized feedback approach on students’ mathematics word problem-solving performance and SRL. Both classes of students took the course guided by the same teacher. The entire experimental process followed a double-blind rule, whereby neither teachers nor participants were informed of the purpose of the experiment in advance. All students had previously completed the information technology courses and had mastered basic software and hardware knowledge. In other words, all the students had similar information technology backgrounds and experience.

**Figure 5:**
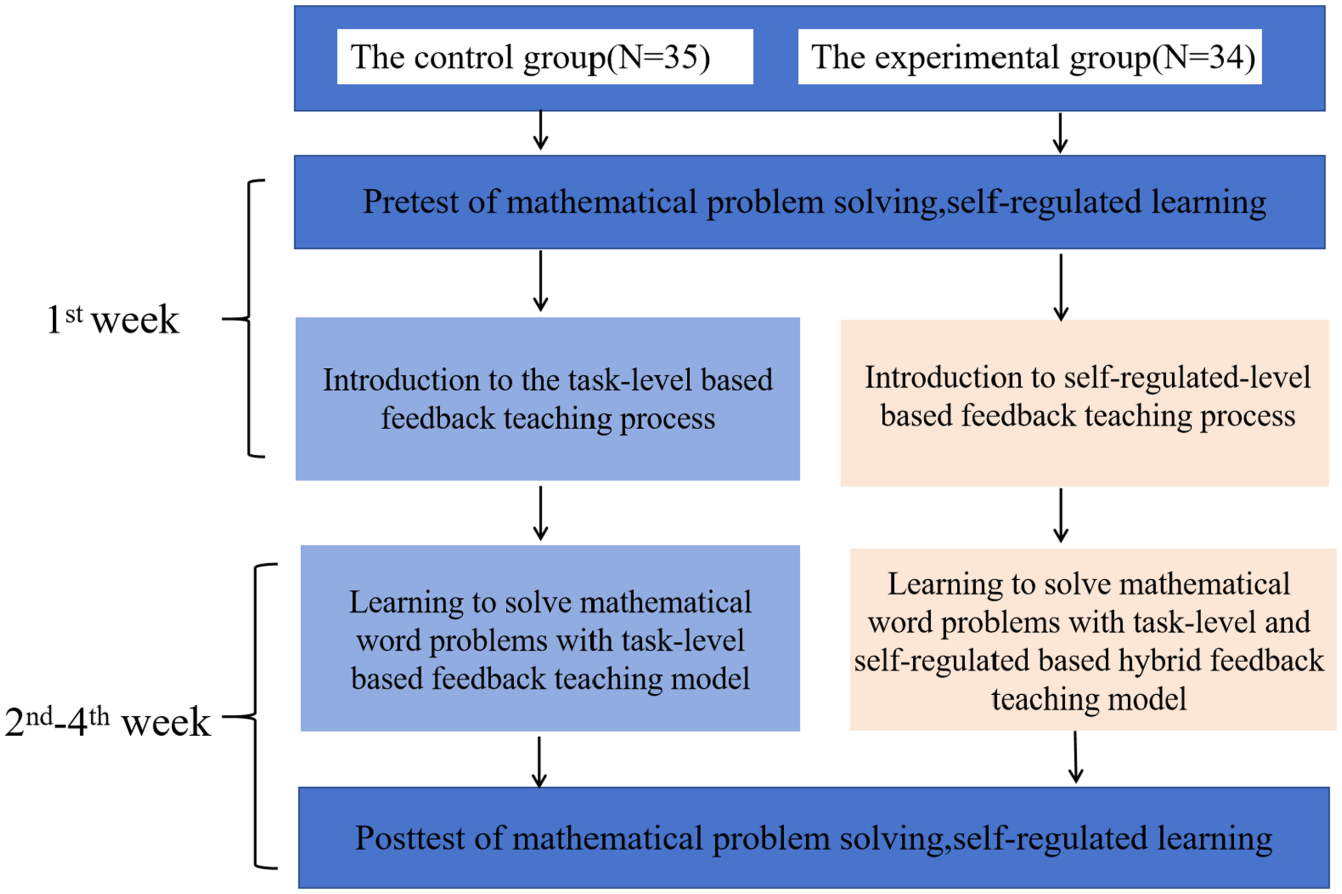
The experimental procedure.

Figure 5 shows the experimental procedure. The whole experiment lasted for 4 weeks with one 35-min lesson per week. In the first week, the students from both groups took the pretest of mathematics word problem solving and the SRL survey. Following that, the teacher introduced the teaching process to the students in both groups. From the second to fourth week, students in the control group learned to solve mathematical word problems with the TLF teaching model, while students in the experimental group learned with the SRLF teaching model to support their mathematics word problem solving. In the fourth week, all of the students took the posttest of mathematics word problem solving and completed the self-report of self-regulated learning.

### 4.3 Instruments

The instruments used in this study involved the pretest and posttest of mathematics word problems, as introduced in 4.3.1, the self-reported SRL questionnaire, as introduced in 4.3.2, and the interactive e-book software, as introduced in 4.3.3.

#### 4.3.1 Mathematics word problems test

The pretest and posttest were developed by two experienced teachers. The pretest aimed to evaluate the students’ prior knowledge of the multiplier unit, and the posttest intended to evaluate the mastery of the multiplier unit. Both tests consisted of six mathematics word problems. The full scores for the pretest and posttest were, respectively, 60. In addition, two experienced mathematics teachers validated the pretest and posttest items. Supplementary Figure 8 shows the test of mathematics word problems for students.

#### 4.3.2 Self-regulated learning questionnaire

The SRL questionnaire was modified from a scale developed by Cavas et al. (2020). It used a 5-point Likert scale (1: *strongly disagree*, 5: *strongly agree*) and consisted of 26 questions, including eight for “academic goal setting,” five for “cognitive strategies,” five for “metacognitive strategies,” three for “intrinsic motivation,” and five for “self-efficacy.” Supplementary Table 1 shows the reliability of this questionnaire. The total Cronbach’s alpha for the questionnaire was 0.886, and the Cronbach’s alpha values for the five dimensions were 0.877, 0.754, 0.729, 0.716 and 0.865, respectively. Therefore, this questionnaire has good reliability. Supplementary Figure 9 shows the SRL questionnaire for students.

#### 4.3.3 Interactive e-book

The interactive e-book platform has such affordances as editing, collection, automatic correction, visualization of data feedback results, and so on. Teachers can edit the lesson content, set web links and distribute curriculum resources to students. Students can return to that page by clicking on the hyperlink and complete dynamic interactive learning on the interactive e-book platform. It can meet the teaching requirements of this experiment and support the web page version for tablet PCs, allowing students to open curriculum resources on their tablet PCs. The student edit interface on this interactive e-book platform is shown in Figure 4.

## 5 Results

This section is divided into three parts: 5.1 offers a comprehensive overview of the data analysis methods utilized in this study. Subsequently, detailed data analysis results of mathematical problem-solving performance are provided in 5.2, while 5.3 provides detailed data analysis results related to SRL (Supplementary Figure 10).

### 5.1 Data analysis

This study used the students’ pretest scores of mathematics word problem solving and self-regulated learning, respectively, as covariates. Before ANCOVA, the homogeneity of covariate regression coefficients was examined to justify the assumption of regression homogeneity for ANCOVA. If satisfied, ANCOVA was conducted to analyze the differences between the experimental group and the control group in mathematics word problem solving and SRL (Supplementary Figure 11).

ANCOVA was conducted where students’ pretest scores were treated as a covariate. First, the assumption of homogeneity of regression was accepted (*F* = 2.711, *p* = 0.104 > 0.005), which indicated that ANCOVA could be used to interpret the effect of the personalized feedback approach on their mathematics word problem-solving performance under the control of the pretest.

In addition, according to the Shapiro–Wilk data normal distribution test, the *p*-values of the pretest of SRL and the five dimensions were 0.120, 0.071, 0.069, 0.134, 0.176, and 0.095, respectively, which satisfy normal distribution. Therefore, in this study, the independent samples *t* test was conducted to analyze the pretests of SRL competence and the five dimensions, with SRL competence and the pretest score of each dimension as the dependent variable and the feedback approach as the grouping variable. The ANCOVA and one-way repeated ANOVA were conducted to analyze the posttest of SRL and the five dimensions.

### 5.2 Effects of the data-driven feedback approach on mathematics word problem-solving performance

ANCOVA results of mathematics word problem-solving performance are shown in Table 2. As for their mathematics word problem-solving performance, the adjusted mean score of the experimental group (42.73) was much higher than that of the control group (37.66), and a statistically significant difference was observed (*F* = 7.91, *p* = 0.006 < 0.005, *η*^2^ = 0.107).

**Table 2:** The ANCOVA results of mathematics word problem-solving performance.

Group	*N*	Pretest *M* (SD)	Posttest *M* (SD)	Adj. *M* (SD)	ANCOVA F *p* η^2^		
EG	35	19.09 (16.39)	43.94 (12.67)	42.73 (1.26)	7.91^**^	0.006	0.107
CG	34	15.06 (11.29)	36.41 (9.78)	37.66 (1.28)			

*p < 0.05, **p < 0.01, ***p < 0.001.

Based on the ANCOVA results, the personalized feedback approach promoted the students’ mathematics word problem-solving performance. In particular, the SRLF approach significantly improved students’ mathematics word problem-solving performance, since it scaffolded students’ SRL, helping them identify difficulties in problem solving, and providing appropriate personalized feedback to improve their mathematics word problem-solving performance.

### 5.3 Effects of the data-driven feedback approach on self-regulated learning

The independent samples *t* test results of the analysis are shown in Supplementary Table 2. No significant difference was found between the two groups on the SRL pretest. In order to measure the differences between the experimental group and the control group on SRL and the five dimensions, we analyzed the pretest and posttest of SRL and the five dimensions of the two groups by using one-way repeated measures variance analysis. The results of the analysis are shown in Supplementary Table 3. With regard to SRL, a statistically significant difference was observed in the time variable. However, there was no significant difference in the time variable for other dimensions. Meanwhile, there was no significant difference in the SRL of the two groups. In other words, both personalized feedback approaches promoted the students’ SRL. Therefore, we analyzed the pretest and posttest changes of the two groups separately using paired-samples *t* tests. The results of the analysis are shown in Supplementary Table 4. According to Supplementary Table 4, there was no significant difference in SRL or in other dimensions for the experimental group. However, for the control group, SRL, cognitive strategies, and metacognitive strategies all significantly improved.

In addition, according to Supplementary Table 3, we found that the interaction between the time and group was more significant for cognitive strategies and SRL. Figure 5 shows the interaction effect between time and group on SRL, and found that there was a significant change in the SRL of the control group compared to the experimental group. Supplementary Figure 8 shows the interaction effect between time and group on cognitive strategies, and found that there was a significant change in the cognitive strategies of the control group compared to the experimental group. In other words, the TLF approach had a greater impact on cognitive strategies and SRL.

In order to further explore the differences between the two feedback approaches in terms of SRL, an analysis of covariance (ANCOVA) was employed using the students’ self-regulated learning pretest scores as a covariate. The assumption of homogeneity of regression was satisfied for ANCOVA (*F* = 1.505, *p* = 0.224 > 0.005). Hence, ANCOVA was used to verify the effect of the personalized feedback approach on students’ SRL under the control of the pretest.

As illustrated in Table 3, there was no statistically significant difference between the two groups (*F* = 2.36, *p* = 0.129 > 0.005), while the adjusted mean score of the experimental group (99.88) was slightly higher than that of the control group (95.31). Meanwhile, from Table 3, there were no significant differences in academic goal setting (*F* = 1.33, *p* = 0.253 > 0.005), cognitive strategies (*F* = 1.35, *p* = 0.250 > 0.005), metacognitive strategies (*F* = 0.93, *p* = 0.337 > 0.005), intrinsic motivation (*F* = 0.63, *p* = 0.430 > 0.005) or self-efficacy (*F* = 1.29, *p* = 0.260 > 0.005). However, the experimental group, respectively, showed slightly higher adjusted mean scores in these five aspects than the control group. Combining the pretest and paired-sample *t*-test analysis results of SRL, the TLF approach was slightly superior to the SRLF approach during the short-term experimentation.

**Table 3:** The ANCOVA results of self-regulated learning.

DV	Group	*N*	Posttest *M* (SD)	Adj. *M* (SD)	ANCOVA		
					*F*	*p*	η^2^
Self-regulated learning	EG	35	101.26 (12.10)	99.88 (2.06)	2.36	0.129	0.035
	CG	34	93.85 (17.88)	95.31 (2.12)			
Academic goal setting	EG	35	28.23 (7.82)	27.48 (0.71)	1.33	0.253	0.020
	CG	34	25.52 (7.86)	26.31 (0.73)			
Cognitive strategies	EG	35	18.71 (3.55)	18.19 (0.43)	1.35	0.250	0.020
	CG	34	16.91 (4.18)	17.47 (0.44)			
Metacognitive strategies	EG	35	20.77 (3.37)	20.31 (0.46)	0.93	0.337	0.014
	CG	34	19.18 (4.38)	19.67 (0.47)			
Intrinsic motivation	EG	35	13.14 (1.94)	13.23 (0.28)	0.63	0.430	0.010
	CG	34	13.00 (2.49)	12.91 (0.29)			
Self-efficacy	EG	35	20.40 (3.54)	20.27 (0.54)	1.29	0.260	0.019
	CG	34	19.24 (4.75)	19.38 (0.56)			

## 6 Discussion and conclusion

Mathematics learning is seen not only as knowledge acquisition but also as student participation in a learning community (Nemati et al., 2020), specifically as it asks students to explain and justify their thinking, and discuss how to effectively use mathematical models in different problem-solving situations (Etheris and Tan, 2004). With the development of information technology, data-driven feedback approaches have attracted widespread attention because they can provide students with precise learning objectives and personalized feedback that promotes their SRL, and can monitor the progress of guiding decision making. In conclusion, although some studies have demonstrated that the personalized feedback approach can improve students’ learning performance and SRL in mathematics, the relationship between different feedback types and learning effectiveness, and the underlying reasons have not as yet been fully explained. This issue still needs to be examined and verified by a large number of studies.

Hence, this section aims to explore: (1) Which type of feedback approach can significantly improve mathematics problem-solving performance: the TLF approach or the SRLF teaching model?; and (2) Which type of feedback approach can significantly improve self-regulated learning: the TLF approach or the SRLF approach? This section presents relevant discussions based on the data analysis results. Based on the discussion, the limitations and future prospects of this study are identified, ultimately leading to well-founded conclusions.

## 7 Discussion

### 7.1 Effects of the feedback-based approach on mathematics word problem solving

The experimental results showed that both feedback approaches promoted students’ math problem-solving scores. This finding is consistent with previous studies which argued that data-driven feedback teaching tools positively affect students’ mathematics performance (Koedinger et al., 2010; Bokhove and Drijvers, 2012; Bulunuz et al., 2014; De Witte et al., 2015). Meanwhile, this result also validated the point of Greene et al. (2018) who conducted a stratified randomized controlled trial to evaluate mathematics addition and subtraction fluency instruction embedded within a feedback teaching framework. Students in the experimental group received multiple interventions, including validating and corrective feedback. The results showed that the experimental group’s academic performance in mathematics significantly improved.

Moreover, the SRLF approach can more significantly stimulate students’ mathematics word problem-solving scores than the TLF approach. Hattie and Timperley (2007) argued that TLF allows learners to focus on the task, such as providing information about the correct answer (e.g., “You explained the limitations, but you could also explain why they are unreliable”). In this case, feedback is powerful if it is more information-focused (e.g., correct or incorrect), leads people to obtain more information or different information, and builds more surface knowledge. It also appears to be more effective when the learner is a novice. The SRLF approach involves the ability to self-evaluate, increase task effort, or seek further feedback (e.g., “What will happen if you increase the temperature in your study?”). At this level of feedback, there will be more information directed at the learner’s self, and more information to guide the learner when and where to use both process-level strategies. In general, it is not that more specific feedback information is more conducive to students’ higher academic performance and SRL ability. In fact, simple feedback may be more effective for novices. The results of this study illustrate exactly this point.

In general, TLF is the basis of SRLF (Hattie and Timperley, 2007). In this study, the experimental group adopted a SRLF approach, which included reflection on the causes of errors and learning scaffolding for the particular erroneous cause. In contrast, the control group adopted a TLF approach, which only involved the accuracy of their answers. Therefore, the experimental group used a feedback instrument that was much more detailed than that used by the control group. There was a significant positive correlation between the frequency of students’ tool use and their learning effectiveness (Faber et al., 2017). In addition, it has been proven that “knowledge of results” feedback (i.e., feedback that provides correct answers) has a less positive effect, whereas detailed feedback has a more positive effect on learning effectiveness (Lipnevich and Smith, 2009; Candel et al., 2021). Specific feedback informs students of which mistakes they made, as well as providing an explanation of the correct answer. Specific feedback can be more effective than simple feedback (knowledge of the results) when students solve complex problems (Candel et al., 2021). Studies on the effectiveness of digital formative assessment algorithms and mathematics tools also verified this opinion (Bokhove and Drijvers, 2012).

### 7.2 Effects of feedback-based approaches on self-regulated learning

The experimental results indicated that both feedback approaches significantly improved students’ SRL. At different levels, the effects of feedback are different. However, the two types of feedback, task-level and self-regulation-level, often give students the most appropriate suggestions for choosing the next step, fostering more self-regulation of the learning process, deepening their own understanding, and obtaining more information (Hattie and Timperley, 2007). This is consistent with Afzaal et al.’s finding that providing intelligent feedback and suggestions in a data-driven manner can improve students’ SRL (Afzaal et al., 2023). It is also consistent with Kim and Hannafi’s finding that feedback teaching strategies in a computer environment are an important method to promote students’ SRL (Kim and Hannafin, 2011).

In addition, this study also found that there was no significant difference between the effects of the two feedback approaches on SRL, but the control group slightly outperformed the experimental group. In other words, the TLF approach had a greater impact on SRL, particularly in terms of SRL and cognitive strategies. Moreover, the feedback type and learning time had significant interaction effects on total scores of SRL and cognitive strategies. This means that in terms of SRL and cognitive strategies, changes throughout the experiment were more significant for the control group, but for the experimental group, the change was unremarkable. The results of this experiment exactly verify Heidi and Timperley’s view, namely that simple feedback may be more effective than complex feedback, especially in terms of SRL ability. Meanwhile, this result is consistent with the studies of Boesen et al. (2014) and Candel et al. (2021). Boesen et al. (2014) pointed out that if problem solving is facilitated by providing TLF, that is, not providing feedback on how to solve the task or the steps, students will be able to think harder and more positively about solutions to mathematics problems when faced with learning tasks, boosting their regulated learning ability. Furthermore, Candel et al. (2021) concluded that digital learning environments may become more effective if the feedback includes a personal diagnostic element, that is, the capacity to provide feedback on whether it is correct or not. In this study, the TLF approach provided feedback on the correctness of mathematical word problem solving, while the SRLF approach provided appropriate personalized feedback based on the students’ causes of error. Students who learned with the TLF were able to think more positively about how to solve the mathematical word problems, which stimulated their SRL competence. Therefore, it explains the larger effect on SRL in the control group that learned with the TLF approach than in the experimental group that learned with the SRLF approach.

### 7.3 Limitations and suggestions

#### 7.3.1 Limitations

This study extends the application of data-driven feedback in classroom teaching, and provides a paradigmatic reference for personalized feedback teaching with data-driven support. However, there are some limitations to this study. First and foremost, the proposed approach was evaluated in only one mathematics course, which limits its generalizability. Second, the experiment lasted for a short-term period, which affected the significant improvement of SRL. Third, this study only explored two types of personalized feedback.

#### 7.3.2 Future work

In our future work, we plan to evaluate the effectiveness of the approach in other courses, such as language reading comprehension. Moreover, it is suggested that the experiment should be conducted for over 3 months. Finally, more alternative types of personalized feedback should be investigated for their effects on academic achievements and SRL.

## 8 Conclusion

(1) This study indicated that both personalized feedback approaches contributed to the improvement of mathematics problem-solving performance, especially the task-level feedback approach. Therefore, this study complements the existing literature on applying SRL to develop students’ mathematics learning performance (Chiesa and Robertson, 2000; Greene et al., 2018; Evans et al., 2021).
(2) Moreover, both personalized feedback approaches contributed to the improvement of SRL ability. Nevertheless, there was an insignificant difference between the two personalized feedback approaches for SRL. The task-level feedback approach had a slightly higher impact on SRL ability than the SRLF approach.

In sum, the findings of this study re-validated that the personalized feedback approach significantly enhanced students’ learning of mathematical concepts and contributed to the development of SRL. In addition, simple feedback has a greater impact on students’ mathematical problem-solving and SRL abilities than complex feedback. This research result can be a reference for subsequent feedback teaching design.

## Data availability statement

The original contributions presented in the study are included in the article/supplementary materials, further inquiries can be directed to the corresponding author/s.

## Ethics statement

This research was reviewed and approved by the Institution Review Board of the Wenzhou University (Code number: WZU-2023-099). The studies were conducted in accordance with the local legislation and institutional requirements. Written informed consent for participation in this study was provided by the participants’ legal guardians/next of kin. Written informed consent was obtained from the individual(s), and minor(s)’ legal guardian/next of kin, for the publication of any potentially identifiable images or data included in this article.

## Author contributions

JH: Writing – review & editing, Writing – original draft, Visualization, Validation, Supervision, Software, Resources, Project administration, Methodology, Investigation, Funding acquisition, Formal analysis, Data curation, Conceptualization. YC: Writing – review & editing, Visualization, Validation, Supervision, Software, Resources, Project administration, Methodology, Investigation, Funding acquisition, Formal analysis, Data curation, Conceptualization. ZL: Writing – review & editing, Software, Investigation, Conceptualization. YH: Writing – review & editing, Writing – original draft. X-LZ: Writing – review & editing, Writing – original draft.

## References

[ref1] Afzaal M. Nouri J. Zia A. Papapetrou P. Fors U. Yongchao W.. (2021). Explainable AI for data-driven feedback and intelligent action recommendations to support students self-regulation. Front. Artif. Intell. 37:723447. doi: 10.3389/frai.2021.723447, PMID: 34870183 PMC8636130

[ref2] Afzaal M. Zia A. Nouri J. Fors U. (2023). Informative feedback and explainable AI-based recommendations to support students’ self-regulation. Technol. Knowl. Learn. 29, 331–354. doi: 10.1007/s10758-023-09650-0

[ref3] Algayres M. Triantafyllou E. (2020). Learning analytics in flipped classrooms: a scoping review. Electron. J. E-Learn. 18:3. doi: 10.34190/JEL.18.5.003

[ref4] Alonso-Mencía M. E. Alario-Hoyos C. Estévez-Ayres I. Kloos C. D. (2021). Analysing self-regulated learning strategies of MOOC learners through self-reported data. Australas. J. Educ. Technol., 56–70. doi: 10.14742/ajet.6150

[ref9001] Artino Jr A. R. Jones II K. D. (2012). Exploring the complex relations between achievement emotions and self-regulated learning behaviors in online learning. The internet and higher education. 15, 170–175. doi: 10.1016/j.iheduc.2012.01.006

[ref5] Bae H. Kwon K. (2021). Developing metacognitive skills through class activities: what makes students use metacognitive skills? Educ. Stud. 47, 456–471. doi: 10.1080/03055698.2019.1707068

[ref6] Balzer W. K. Doherty M. E. O'Connor R. Jr. (1989). Effects of cognitive feedback on performance. Psychol. Bulletin 106, 410–433. doi: 10.1037/0033-2909.106.3.410

[ref7] Baneres D. Elena Rodriguez M. Serra M. (2019). An early feedback prediction system for learners at-risk within a first-year higher education course. IEEE Trans. Learn. Technol. 12, 249–263. doi: 10.1109/TLT.2019.2912167

[ref8] Black P. Wiliam D. (1998). Assessment and classroom learning. Assess. Educ. 5, 7–74. doi: 10.1080/0969595980050102

[ref9] Boesen J. Helenius O. Bergqvist E. Bergqvist T. Lithner J. Palm T.. (2014). Developing mathematical competence: from the intended to the enacted curriculum. J. Math. Behav. 33, 72–87. doi: 10.1016/j.jmathb.2013.10.001

[ref10] Bokhove C. Drijvers P. (2012). Effects of a digital intervention on the development of algebraic expertise. Comput. Educ. 58, 197–208. doi: 10.1016/j.compedu.2011.08.010

[ref11] Bulunuz N. Bulunuz M. Peker H. (2014). Effects of formative assessment probes integrated in extra-curricular hands-on SCIENCE: middle school students’ understanding. J. Balt. Sci. Educ. 13, 243–258. doi: 10.33225/jbse/14.13.243

[ref12] Butler D. Winne P. (1995). Feedback and self-regulated learning: a theoretical synthesis. Rev. Educ. Res. 65, 245–281. doi: 10.3102/00346543065003245

[ref13] Candel C. Máñez I. Cerdán R. Vidal-Abarca E. (2021). Delaying elaborated feedback within computer-based learning environments: the role of summative and question-based feedback. J. Comput. Assist. Learn. 37, 1015–1029. doi: 10.1111/jcal.12540

[ref14] Cano A. Leonard J. D. (2019). Interpretable Multiview early warning system adapted to underrepresented student populations. IEEE Trans. Learn. Technol. 12, 198–211. doi: 10.1109/TLT.2019.2911079

[ref15] Cavanagh T. Chen B. Lahcen R. A. M. Paradiso J. (2020). Constructing a design framework and pedagogical approach for adaptive learning in higher education: a Practitioner’s perspective. Int. Rev. Res. Open Distributed Learn. 21, 172–196. doi: 10.19173/irrodl.v21i1.4557

[ref16] Cavas P. Arslan-Cansever B. Ünver G. (2020). Developing the perceived self-regulation skills scale for fourth grade students. Croatian J. Educ. 22:3623. doi: 10.15516/cje.v22i3.3623

[ref17] Chen C.-H. Chien-Yuan S. (2019). Using the BookRoll E-book system to promote self-regulated learning, self-efficacy and academic achievement for university students. J. Educ. Technol. 22, 33–46. doi: 10.2307/26910183

[ref18] Chen P. P. Zimmerman B. J. (2007). A cross-national comparison study on the accuracy and predictability of the self-efficacy beliefs of middle-school mathematics students. J. Exp. Educ. 75, 221–244. doi: 10.3200/JEXE.75.3.221-244

[ref19] Chiesa M. Robertson A. (2000). Precision teaching and fluency training: making Maths easier for pupils and teachers. Educ. Psychol. Pract. 16, 297–310. doi: 10.1080/713666088

[ref20] Copur-Gencturk Y. Doleck T. (2021). Strategic competence for multistep fraction word problems: an overlooked aspect of mathematical knowledge for teaching. Educ. Stud. Math. 107, 49–70. doi: 10.1007/s10649-021-10028-1

[ref21] Daniela P. (2015). The relationship between self-regulation, motivation and performance at secondary school students. Procedia. Soc. Behav. Sci. 191, 2549–2553. doi: 10.1016/j.sbspro.2015.04.410

[ref22] De Witte K. Haelermans C. Rogge N. (2015). The effectiveness of a computer-assisted math learning program. J. Comput. Assist. Learn. 31, 314–329. doi: 10.1111/jcal.12090

[ref23] Demssie Y. N. Biemans H. J. A. Wesselink R. Mulder M. (2023). Fostering students’ systems thinking competence for sustainability by using multiple real-world learning approaches. Environ. Educ. Res. 29, 261–286. doi: 10.1080/13504622.2022.2141692

[ref24] Devolder A. Van Braak J. Tondeur J. (2012). Supporting self-regulated learning in computer-based learning environments: systematic review of effects of scaffolding in the domain of Science education. J. Comput. Assist. Learn. 28, 557–573. doi: 10.1111/j.1365-2729.2011.00476.x

[ref25] Dignath C. Büttner G. (2008). Components of fostering self-regulated learning among students. A meta-analysis on intervention studies at primary and secondary school level. Metacogn. Learn. 3, 231–264. doi: 10.1007/s11409-008-9029-x

[ref26] Etheris A. I. Tan S. C. (2004). Computer-supported collaborative problem solving and anchored instruction in a mathematics classroom: an exploratory study. Int. J. Learn. Technol. 1:16. doi: 10.1504/ijlt.2004.003680

[ref27] Evans A. L. Bulla A. J. Kieta A. R. (2021). The precision teaching system: a synthesized definition, concept analysis, and process. Behav. Anal. Pract. 14, 559–576. doi: 10.1007/s40617-020-00502-2, PMID: 33425240 PMC7781427

[ref28] Faber J. M. Luyten H. Visscher A. J. (2017). The effects of a digital formative assessment tool on mathematics achievement and student motivation: results of a randomized experiment. Comput. Educ. 106, 83–96. doi: 10.1016/j.compedu.2016.12.001

[ref29] Garcia R. Falkner K. Vivian R. (2018). Systematic literature review: self-regulated learning strategies using e-learning tools for computer Science. Comput. Educ. 123, 150–163. doi: 10.1016/j.compedu.2018.05.006

[ref30] Gaul C. Kim M. K. (2020). Learner participation regulation supported by long-term peer moderation and participation feedback during asynchronous discussions. J. Comput. Educ. 7, 295–331. doi: 10.1007/s40692-020-00158-5

[ref31] Greene J. A. Copeland D. Z. Deekens V. M. Seung B. Y. (2018). Beyond knowledge: examining digital Literacy’s role in the Acquisition of Understanding in Science. Comput. Educ. 117, 141–159. doi: 10.1016/j.compedu.2017.10.003

[ref32] Guo W. (2020). Grade-level differences in teacher feedback and students’ self-regulated learning. Front. Psychol. 11:783. doi: 10.3389/fpsyg.2020.00783, PMID: 32431642 PMC7214680

[ref33] Guo W. Lau K. L. Wei J. (2019). Teacher feedback and students’ self-regulated learning in mathematics: a comparison between a high-achieving and a low-achieving secondary schools. Stud. Educ. Eval. 63, 48–58. doi: 10.1016/j.stueduc.2019.07.001

[ref34] Harskamp E. G. Suhre C. J. M. (2006). Improving mathematical problem solving: a computerized approach. Comput. Hum. Behav. 22, 801–815. doi: 10.1016/j.chb.2004.03.023

[ref35] Hasibuan A. M. Saragih S. Amry Z. (2018). Development of learning materials based on realistic mathematics education to improve problem solving ability and student learning Independence. Int. Electron. J. Math. Educ. 14:4000. doi: 10.29333/iejme/4000

[ref36] Hattie J. Timperley H. (2007). Reviewed work(s): “The power of feedback”. Rev. Educ. Res. 77, 81–112. doi: 10.3102/003465430298487

[ref37] Herbert S. Williams G. (2023). Eliciting mathematical reasoning during early primary problem solving. Math. Educ. Res. J. 35, 77–103. doi: 10.1007/s13394-021-00376-9

[ref38] Hwang G.-J. Xie H. Wah B. W. Gasevic D. (2020). Vision, challenges, roles and research issues of artificial intelligence in education. Comput. Educ. Artif. Intell. 1:100001. doi: 10.1016/j.caeai.2020.100001

[ref39] Kabudi T. Pappas I. Olsen D. H. (2021). AI-enabled adaptive learning systems: a systematic mapping of the literature. Comput. Educ. 2:100017. doi: 10.1016/j.caeai.2021.100017

[ref40] Kapoor G. Vostanis A. Mejía-Buenaño S. Langdon P. E. (2023). Using precision teaching to improve typically developing Student’s mathematical skills via teleconferencing. J. Behav. Educ., 1–30. doi: 10.1007/s10864-023-09520-w, PMID: 37359174 PMC10204687

[ref41] Kim M. C. Hannafin M. J. (2011). Scaffolding problem solving in technology-enhanced learning environments (TELEs): bridging research and theory with practice. Comput. Educ. 56, 403–417. doi: 10.1016/j.compedu.2010.08.024

[ref42] Kizilcec R. F. Pérez-Sanagustín M. Maldonado J. J. (2017). Self-regulated learning strategies predict learner behavior and goal attainment in massive open online courses. Comput. Educ. 104, 18–33. doi: 10.1016/j.compedu.2016.10.001

[ref43] Kluger A. N. DeNisi A. (1996). The effects of feedback interventions on performance: a historical review, a meta-analysis, and a preliminary feedback intervention theory. Psychol. Bull. 119, 254–284. doi: 10.1037/0033-2909.119.2.254

[ref44] Koedinger K. R. McLaughlin E. A. Heffernan N. T. (2010). A quasi-experimental evaluation of an on-line formative assessment and tutoring system. J. Educ. Comput. Res. 43, 489–510. doi: 10.2190/EC.43.4.d

[ref45] Labuhn A. S. Bögeholz S. Hasselhorn M. (2008). Lernförderung Durch Anregung Der Selbstregulation Im Naturwissenschaftlichen Unterricht. Zeitschrift für Pädagogische Psychologie 22, 13–24. doi: 10.1024/1010-0652.22.1.13

[ref46] Labuhn A. S. Zimmerman B. J. Hasselhorn M. (2010). Enhancing students’ self-regulation and mathematics performance: the influence of feedback and self-evaluative standards. Metacogn. Learn. 5, 173–194. doi: 10.1007/s11409-010-9056-2

[ref47] Li W.-T. Shein P. P. (2022). Developing sense of place through a place-based indigenous education for sustainable development curriculum. Environ. Educ. Res. 29, 692–714. doi: 10.1080/13504622.2022.2098933

[ref48] Lipnevich A. A. Smith J. K. (2009). Effects of differential feedback on students’ examination performance. J. Exp. Psychol. Appl. 15, 319–333. doi: 10.1037/a0017841, PMID: 20025418

[ref49] Maier U. Klotz C. (2022). Personalized feedback in digital learning environments: classification framework and literature review. Comput. Educ. Artif. Intel. 3:100080. doi: 10.1016/j.caeai.2022.100080

[ref50] Manzanares S. Consuelo M. Sánchez R. M. Osorio C. I. G. Díez-Pastor J. F. (2017). How do B-learning and learning patterns influence learning outcomes? Front. Psychol. 8:745. doi: 10.3389/fpsyg.2017.00745, PMID: 28559866 PMC5432653

[ref51] Mao P. Cai Z. Wang Z. Hao X. Fan X. Sun X. (2024). The effects of dynamic and static feedback under tasks with different difficulty levels in digital game-based learning. Internet High. Educ. 60:100923. doi: 10.1016/j.iheduc.2023.100923

[ref52] Masingila J. O. Olanoff D. Kimani P. M. (2018). Mathematical knowledge for teaching teachers: knowledge used and developed by mathematics teacher educators in learning to teach via problem solving. J. Math. Teach. Educ. 21, 429–450. doi: 10.1007/s10857-017-9389-8

[ref53] Mayfield K. H. Chase P. N. (2002). The effects of cumulative practice on mathematics problem solving. J. Appl. Behav. Anal. 35, 105–123. doi: 10.1901/jaba.2002.35-105, PMID: 12102132 PMC1284369

[ref54] Michalsky T. Schechter C. (2013). Preservice teachers’ capacity to teach self-regulated learning: integrating learning from problems and learning from successes. Teach. Teach. Educ. 30, 60–73. doi: 10.1016/j.tate.2012.10.009

[ref55] Mubarak A. A. Cao H. Zhang W. (2022). Prediction of students’ early dropout based on their interaction logs in online learning environment. Interact. Learn. Environ. 30, 1414–1433. doi: 10.1080/10494820.2020.1727529

[ref56] Muis K. R. Psaradellis C. Chevrier M. Di Leo I. Lajoie S. P. (2016). Learning by preparing to teach: fostering self-regulatory processes and achievement during complex mathematics problem solving. J. Educ. Psychol. 108, 474–492. doi: 10.1037/edu0000071

[ref57] Murphy R. F. (2019). Artificial intelligence applications to support K–12 teachers and teaching: A review of promising applications, challenges, and risks. Santa Monica, CA: RAND Corporation.

[ref58] Narciss S. Proske A. Koerndle H. (2007). Promoting self-regulated learning in web-based learning environments. Comput. Hum. Behav. 23, 1126–1144. doi: 10.1016/j.chb.2006.10.006

[ref59] Nemati P. Gawrilow C. Nuerk H.-C. Kühnhausen J. (2020). Self-regulation and mathematics performance in German and Iranian students of more and less math-related fields of study. Front. Psychol. 11:489371. doi: 10.3389/fpsyg.2020.489371, PMID: 33192754 PMC7661690

[ref60] Nicol D. (2010). From monologue to dialogue: improving written feedback processes in mass higher education. Assess. Eval. High. Educ. 35, 501–517. doi: 10.1080/02602931003786559

[ref61] Nicolaou C. T. Korfiatis K. Evagorou M. Constantinou C. (2009). Development of decision-making skills and environmental concern through computer-based, Scaffolded learning activities. Environ. Educ. Res. 15, 39–54. doi: 10.1080/13504620802567007

[ref62] Orosco M. J. Lee Swanson H. O’Connor R. Lussier C. (2013). The effects of dynamic strategic math on English language learners’ word problem solving. J. Spec. Educ. 47, 96–107. doi: 10.1177/0022466911416248

[ref63] Öztürk M. Akkan Y. Kaplan A. (2020). Reading comprehension, mathematics self-efficacy perception, and mathematics attitude as correlates of students’ non-routine mathematics problem-solving skills in Turkey. Int. J. Math. Educ. Sci. Technol. 51, 1042–1058. doi: 10.1080/0020739X.2019.1648893

[ref64] Panadero E. Järvelä S. (2015). Socially shared regulation of learning: a review. Eur. Psychol. 20, 190–203. doi: 10.1027/1016-9040/a000226

[ref65] Panaoura A. (2012). Improving problem solving ability in mathematics by using a mathematical model: a computerized approach. Comput. Hum. Behav. 28, 2291–2297. doi: 10.1016/j.chb.2012.06.036

[ref66] Petrovic J. Pale P. Jeren B. (2017). Online formative assessments in a digital signal processing course: effects of feedback type and content difficulty on students learning achievements. Educ. Inf. Technol. 22, 3047–3061. doi: 10.1007/s10639-016-9571-0

[ref67] Ramey D. Lydon S. Healy O. McCoy A. Holloway J. Mulhern T. (2016). A systematic review of the effectiveness of precision teaching for individuals with developmental disabilities. Rev. J. Autism Dev. Disord. 3, 179–195. doi: 10.1007/s40489-016-0075-z

[ref68] Schoppek W. Tulis M. (2010). Enhancing arithmetic and word-problem solving skills efficiently by individualized computer-assisted practice. J. Educ. Res. 103, 239–252. doi: 10.1080/00220670903382962

[ref69] Schunk D. H. (2005). Self-regulated learning: the educational legacy of Paul R. Pintrich. Educ. Psychol. 40, 85–94. doi: 10.1207/s15326985ep4002_3

[ref70] Schunk D. H. Zimmerman B. J. (2007). Influencing Children’s self-efficacy and self-regulation of Reading and writing through modeling. Read. Writ. Q. 23, 7–25. doi: 10.1080/10573560600837578

[ref71] Shams C. (2023). Students' perceptions of instructor feedback relating to self-regulation in an asynchronous community college course: A qualitative case study (Doctoral dissertation). Harrison, NY: Manhattanville College.

[ref72] Sherafati N. Mahmoudi Largani F. (2023). The potentiality of computer-based feedback in fostering EFL learners’ writing performance, self-regulation ability, and self-efficacy beliefs. J. Comput. Educ. 10, 27–55. doi: 10.1007/s40692-022-00221-3

[ref73] Sleeman W. C. IV Krawczyk B. (2021). Multi-class imbalanced big data classification on spark. Knowl.-Based Syst. 212:106598. doi: 10.1016/j.knosys.2020.106598

[ref74] Söderström S. (2023). Computer-based formative assessment for problem solving. Int. J. Math. Educ. Sci. Technol., 1–25. doi: 10.1080/0020739X.2023.2178982

[ref75] Sung Y.-T. Liao C.-N. Chang T.-H. Chen C.-L. Chang K.-E. (2016). The effect of online summary assessment and feedback system on the summary writing on 6th graders: the LSA-based technique. Comput. Educ. 95, 1–18. doi: 10.1016/j.compedu.2015.12.003

[ref76] Teng F. (2020). Tertiary-level students’ English writing performance and metacognitive awareness: a group metacognitive support perspective. Scand. J. Educ. Res. 64, 551–568. doi: 10.1080/00313831.2019.1595712

[ref77] Tian Y. Fang Y. Li J. (2018). The effect of metacognitive knowledge on mathematics performance in self-regulated learning framework—multiple mediation of self-efficacy and motivation. Front. Psychol. 9:2518. doi: 10.3389/fpsyg.2018.02518, PMID: 30631293 PMC6315178

[ref78] Timmers C. F. Walraven A. Veldkamp B. P. (2015). The effect of regulation feedback in a computer-based formative assessment on information problem solving. Comput. Educ. 87, 1–9. doi: 10.1016/j.compedu.2015.03.012

[ref79] Timmons K. Pelletier J. Corter C. (2016). Understanding Children’s self-regulation within different classroom contexts. Early Child Dev. Care 186, 249–267. doi: 10.1080/03004430.2015.1027699

[ref80] Vasalou A. Benton L. Ibrahim S. Sumner E. Joye N. Herbert E. (2021). Do children with reading difficulties benefit from instructional game supports? Exploring children’s attention and understanding of feedback. Br. J. Educ. Technol. 52, 2359–2373. doi: 10.1111/bjet.13145

[ref81] Verschaffel L. Schukajlow S. Star J. Van Dooren W. (2020). Word problems in mathematics education: a survey. ZDM 52, 1–16. doi: 10.1007/s11858-020-01130-4

[ref82] Verschaffel L. Van Dooren W. Greer B. Mukhopadhyay S. (2010). Reconceptualising word problems as exercises in mathematical modelling. J. Math.-Didakt. 31, 9–29. doi: 10.1007/s13138-010-0007-x

[ref83] Vilkova K. (2022). The promises and pitfalls of self-regulated learning interventions in MOOCs. Technol. Knowl. Learn. 27, 689–705. doi: 10.1007/s10758-021-09580-9

[ref84] Vondrová N. (2022). The effect of an irrelevant number and language consistency in a word problem on pupils’ achievement and reasoning. Int. J. Math. Educ. Sci. Technol. 53, 807–826. doi: 10.1080/0020739X.2020.1782497

[ref85] Wisniewski B. Zierer K. Hattie J. (2020). The power of feedback revisited: a meta-analysis of educational feedback research. Front. Psychol. 10:3087. doi: 10.3389/fpsyg.2019.03087, PMID: 32038429 PMC6987456

[ref86] Xu W. Jun Meng S. Kanaga Raja M. Priya P. Kiruthiga Devi M. (2021). Artificial intelligence in constructing personalized and accurate feedback Systems for Students. Int. J. Model. Simulat. Sci. Comput. 14:15. doi: 10.1142/s1793962323410015

[ref87] Yang T.-C. Chen M. C. Chen S. Y. (2018). The influences of self-regulated learning support and prior knowledge on improving learning performance. Comput. Educ. 126, 37–52. doi: 10.1016/j.compedu.2018.06.025

[ref88] Yang C. C. Y. Ogata H. (2023). Personalized learning analytics intervention approach for enhancing student learning achievement and behavioral engagement in blended learning. Educ. Inf. Technol. 28, 2509–2528. doi: 10.1007/s10639-022-11291-2

[ref89] Yang L. F. Zhang L. J. (2023). Self-regulation and student engagement with feedback: the case of Chinese EFL student writers. J. Engl. Acad. Purp. 63:101226. doi: 10.1016/j.jeap.2023.101226

[ref90] Zepeda C. D. Ortegren F. R. Butler A. C. (2023). Learning from feedback in college courses: student beliefs, practices, and preferences. Appl. Cogn. Psychol. 37, 1238–1257. doi: 10.1002/acp.4118

[ref91] Zimmerman B. J. (2002). Becoming a self-regulated learner: an overview. Theory Pract. 41, 64–70. doi: 10.1207/s15430421tip4102_2

[ref92] Zimmerman B. J. (2008). Investigating self-regulation and motivation: historical background, methodological developments, and future prospects. Am. Educ. Res. J. 45, 166–183. doi: 10.3102/0002831207312909

