# Supplementary material for: Toward self-regulated learning: effects of different types of data-driven feedback on pupils’ mathematics word problem-solving performance
Source: Front Psychol. 2024 Oct 1;15:1356852. doi: 10.3389/fpsyg.2024.1356852 (PMC11473304; doi:10.3389/fpsyg.2024.1356852)
Supplement: Supplementary file 1 [file Presentation_1.pdf]

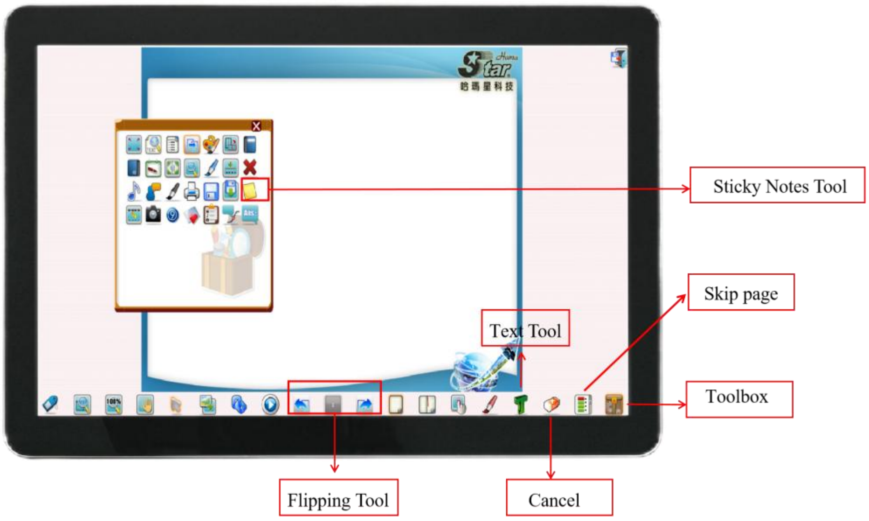


**Supplementary figure 1**. The student edit interface

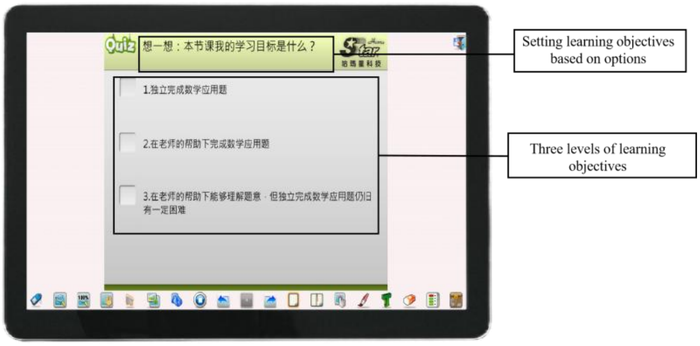


**Supplementary figure 2**. The student interface for setting learning goals

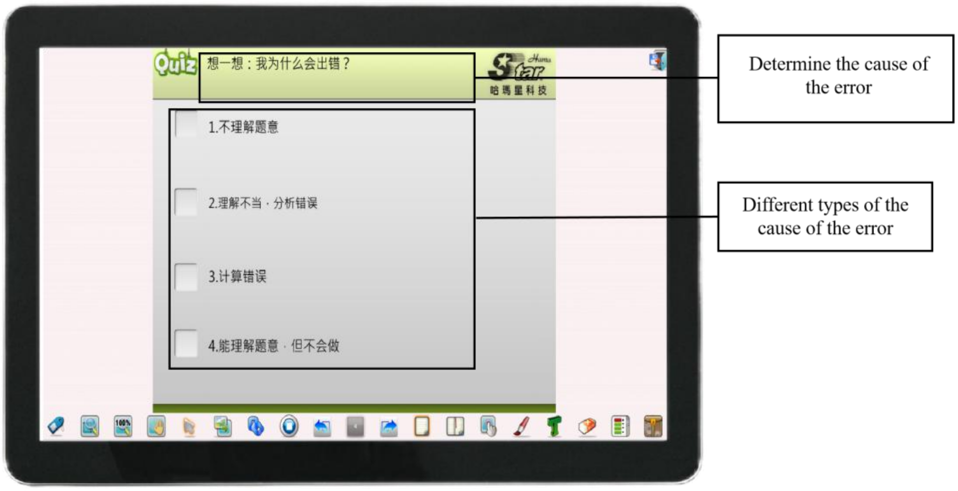


**Supplementary figure 3**. The interface for cause feedback

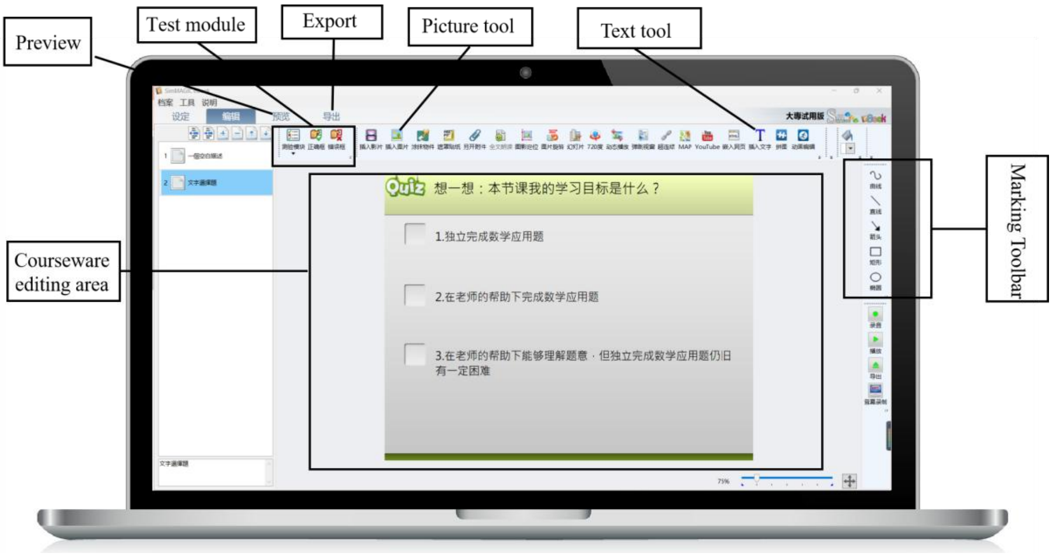


**Supplementary figure 4.** The teacher edit interface

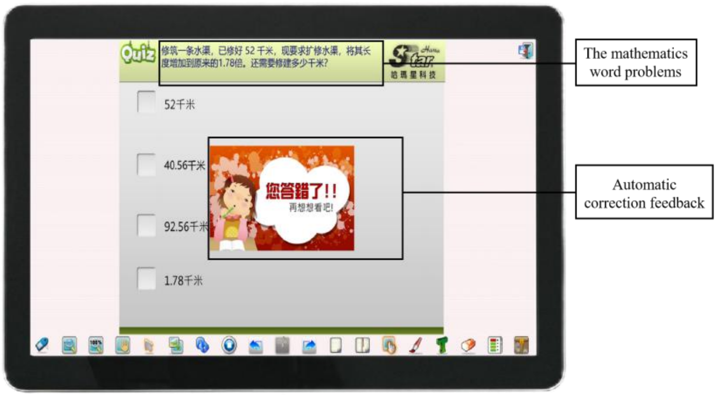


**Supplementary figure 5.** The feedback interface of automatic correction

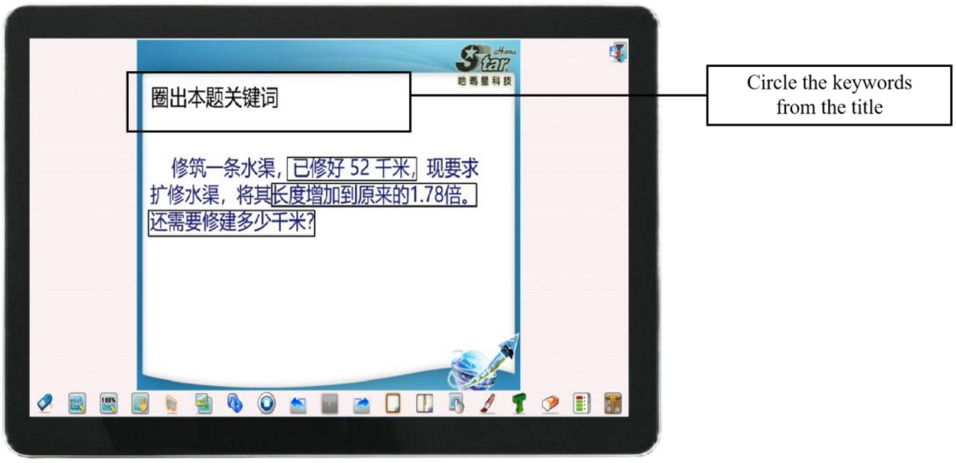


**Supplementary figure 6.** The feedback interface of direction

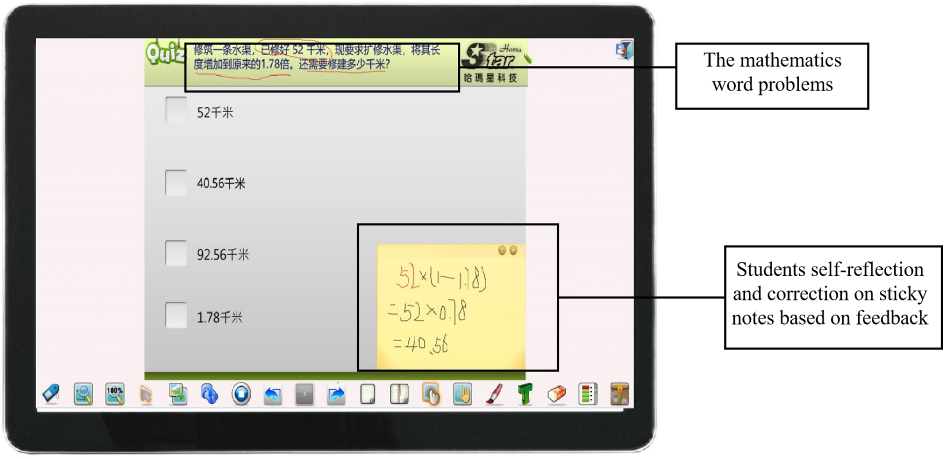


**Supplementary figure 7.** The students' self-reflection-based feedback activities on the interactive e-book platform

Class: Student ID: Name:

1. A Supermarket shipped a total of 300 bags of rice and flour. Among them, the number of bags of flour was 1.5 times that of rice. How many bags of rice and flour were shipped?

2. There is a lawn in a park with an area of 420 square meters. It is now required to expand the lawn to increase its area to the original area.

1.6 times. How many square metres of lawn do I need to build?

3. The perimeter of a rectangle is 15 cm, and the length is twice as long as the width. What is the area of this rectangle in square centimetres?

4. A car travels 110 kilometres per hour. It takes 5 hours to get from point A to point B. The time it takes to return is

It increased by 1.2 times when it came. What is the speed of return?

5. There are 20 people in a chorus. If one person is notified every minute by phone, how long will it take, at least, to notify everyone?

6. There is an emergency announcement in the school that requires the teacher to call the students to inform them. One student will be notified in 1 minute. How many students can be informed at most in 5 minutes?

**Supplementary figure 8.** The mathematics word problems test

Tips:Fill in the following questionnaire according to your own situation,where 1 means totallydisagree and 5 means totally agree.

Age: Student ID: Name: Sex: Number:

1. It's important for me to do better than the other students.
2. I don't want people to think that I'm a terrible student.
3. It's important for me to make my classmates feel that I'm good at learning.
4. It is important for me to be recognized by other students in this class for my academic abilities.
5. I'm worried about being blamed for my poor grades.
6. If I did do better than most of the other students,I would be very proud of myself.
7. I'm willing to show my teacher that I'm better than the other students.
8. One of the main reasons I'm studying is because I don't want to be ashamed of myself because I'm poor at studying.
9. I use drawing diagrams and lists to organize what I have leamed.
10. I organize my leaming activities according to the learning objectives I set for myself.
11. I will make a list of important points to memorize.
12. I will seek help from my classmates when I encounter difficulties in my studies.
13. I often explained mathematical word problem to my classmates.
14. I try to change the way I learn to fit the teacher's teaching style and the requirements of the course.
15. When reading a text,I look for what I should know based on the topic of the text,rather than just reading it.
16. I often skim through a new text to see how it is organized before studying it systematically.
17. I make sure I understand what I'm learning by asking myself questions.
18. I prefer to learn things that arouse my curiosity,even if they are difficult to learn.
19. Learning satisfies my interest in exploring knowledge.
20. I often ask myself questions to help me focus when reading a text.
21. I am interested in the specifics of a particular discipline.
22. I believe I can understand the content of the lessons.
23. I am confident that I can successfully use what I have learned to solve problems.
24. I think I can overcome all difficulties in the learning process and achieve good grades.

**Supplementary figure 9.** Self-Regulated Learning Questionnaire

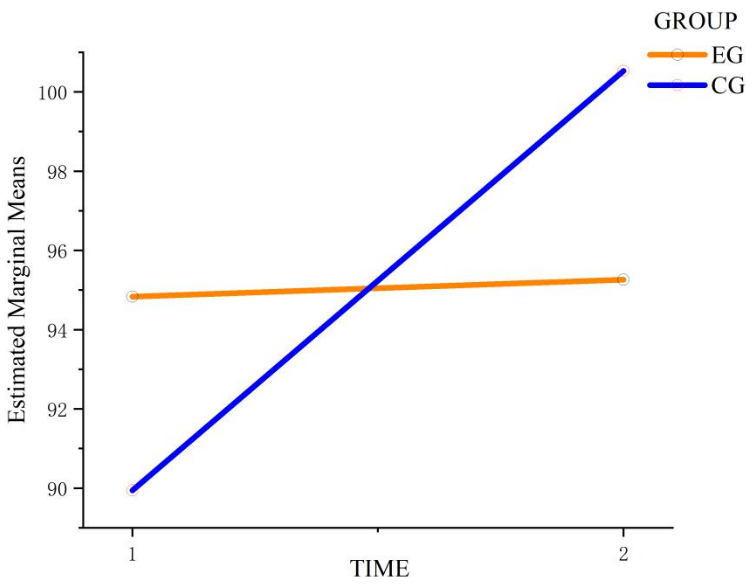


**Supplementary figure 10.** The interaction effect between time and group on self-regulated learning

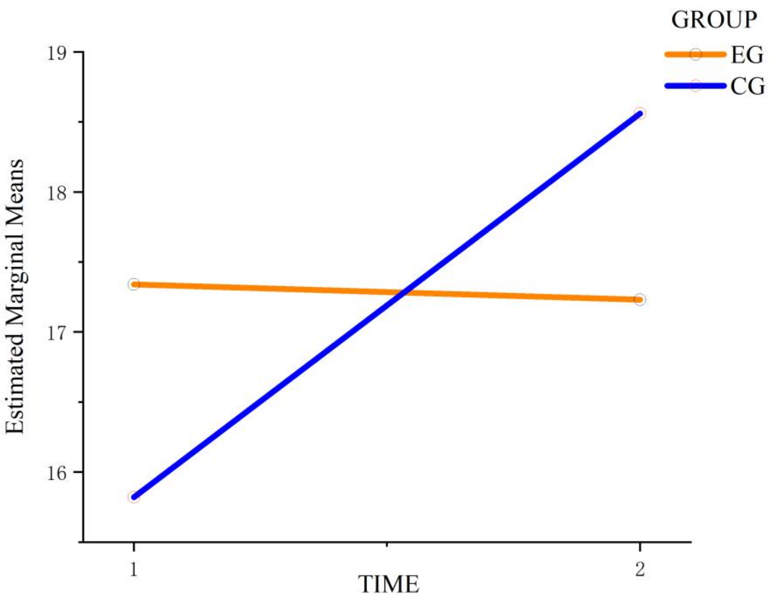


**Supplementary figure 11.** The interaction effect between time and group on cognitive strategy

**Supplementary table 1.** The reliability of the self-regulated learning questionnaire

| Subscales | Cronbach α |
|---|---|
| self-regulated learning | .886 |

| | |
|---|---|
| academic goal setting | .877 |
| cognitive strategies | .754 |
| metacognitive strategies | .729 |
| intrinsic motivation | .716 |
| self-efficacy | .865 |

**Supplementary table 2.** Independent samples *t*-test results for the pretest of self-regulated learning

| DV | GROUP | *N* | *M*(SD) | *t* | *p* |
|---|---|---|---|---|---|
| Self-regulated learning | EG | 35 | 94.83(13.69) | 1.227 | .224 |
| | CG | 34 | 89.94(19.04) | | |
| Academic goal setting | EG | 35 | 26.11(8.35) | .939 | .351 |
| | CG | 34 | 24.24(8.28) | | |
| Cognitive strategies | EG | 35 | 17.34(4.08) | 1.467 | .147 |
| | CG | 34 | 15.82(4.52) | | |
| Metacognitive strategies | EG | 35 | 19.69(3.62) | 1.289 | .202 |
| | CG | 34 | 18.38(4.72) | | |
| Intrinsic motivation | EG | 35 | 12.43(2.52) | .395 | .694 |
| | CG | 34 | 12.68(2.68) | | |
| Self-efficacy | EG | 35 | 19.26(4.08) | .393 | .696 |
| | CG | 34 | 18.82(5.05) | | |

**Supplementary table 3.** Results of one-way repeated ANOVA results of self-regulated learning

| DV | TIME | | | GROUP | | | TIME*GROUP | | |
|---|---|---|---|---|---|---|---|---|---|
| | *F* | *P* | $\eta^2$ | *F* | *P* | $\eta^2$ | *F* | *P* | $\eta^2$ |
| Self-regulated learning | 4.91* | .030 | .068 | .004 | .948 | <.001 | 4.18* | .045 | .059 |
| Academic goal setting | 1.59 | .211 | .023 | <.001 | .984 | <.001 | 1.70 | .197 | .025 |
| Cognitive strategies | 3.51 | .065 | .050 | .018 | .893 | <.001 | 4.15* | .046 | .058 |
| Metacognitive strategies | 2.29 | .135 | .033 | <.001 | .999 | <.001 | 3.98 | .050 | .056 |
| Intrinsic motivation | 1.80 | .184 | .026 | .087 | .769 | .001 | .095 | .759 | .001 |
| Self-efficacy | 1.48 | .228 | .022 | .059 | .809 | .001 | .74 | .392 | .011 |

*$p < .05$, *** $p < .001$.

**Supplementary table 4.** Paired-samples *t*-test results for self-regulated learning

| DV | GROUP | *t* | *p* |
|---|---|---|---|
| Self-regulated learning | EG | -.135 | .894 |
| | CG | -2.759** | .009 |
| Academic goal setting | EG | .030 | .976 |
| | CG | -1.701 | .098 |
| Cognitive strategies | EG | .113 | .910 |
| | CG | -2.829** | .008 |
| Metacognitive strategies | EG | .339 | .737 |
| | CG | -2.492 | .018 |
| Intrinsic motivation | EG | -1.145 | .260 |
| | CG | -.748 | .460 |
| Self-efficacy | EG | -.247 | .807 |
| | CG | -1.508 | .141 |

**$p < .01$.
